# Directed aromatic functionalization

**DOI:** 10.3762/bjoc.7.141

**Published:** 2011-09-06

**Authors:** Victor Snieckus

**Affiliations:** 1 Department of Chemistry, Queen's University, Kingston, Ontario, K7L 3N6, Canada

The title of this Thematic Series brings to the minds of most organic chemists the beautifully logical aromatic electrophilic substitution (S_E_Ar) [1–5] and, to a lesser extent, nucleophilic aromatic substitution (S_N_Ar) [2,6–7] reactions as taught to many generations of students in their first organic chemistry courses [8] (Figure 1). Being less steeped in history, radical nucleophilic substitution (S_RN_1) [9] and vicarious nucleophilic substitution (VNS) [10–12], in spite of their considerable synthetic utility, are given sparse mention. By the time students reach the upper years of study, aromatic chemistry receives the label “classical” and is disregarded or relegated to brief coverage. Apart from in English schools [13], heteroaromatic chemistry suffers the same fate. As a consequence, a fresh graduate entering the pharmaceutical industry, who invariably faces a complex aromatic or heteroaromatic as his or her first target molecule, is either lost or must grope around with insufficient background knowledge.

**Figure 1:**
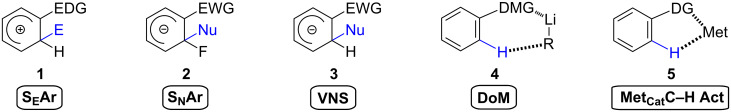
Directed aromatic functionalization methods.

Since the late 1970s, over thirty years since the independent discoveries by Gilman and Wittig, the directed *ortho* metalation (DoM) reaction has trickled into the armamentarium of the synthetic chemist (but not significantly into textbooks [8,14]), as a general and rational strategy for the construction of polysubstituted aromatics and heteroaromatics [15–17]. While comparison with S_E_Ar and S_N_Ar should never be denied, the DoM approach offers incontestable *ortho* regioselectivity, mild conditions, and perhaps most significantly, broad post-DoM synthetic potential. As a result, it has been called upon, with increasing favor and frequency, by academic and medicinal chemists for small-scale synthesis and by process chemists for multi-kilogram scale routes for clinical candidates and commercial pharmaceuticals and agrochemicals [18–24].

Once an aryl metal species was available by DoM and metal–halogen exchange or metal insertion, the gates of the synthetic arena were spectacularly opened to the 2010 Nobel Prize chemistry and the general theme of transition metal-catalyzed reactions. Thus, the named reactions of Kumada–Corriu, Negishi, Suzuki–Miyaura, Stille and, most recently, Hiyama have all given new insights into how sp, sp^2^, and sp^3^, and –O, –N, and –S bonds are made to the aromatic ring carbon [25]. These strategies rapidly furnish biaryls/heterobiaryls, and condensed complex aromatics/heteroaromatics whose construction by previous generation methods (e.g., diazonium and radical coupling) has been clearly superseded by these venerable reactions [26–27] especially to the benefit of the medicinal and process chemist [28].

The currently lively area of metal-catalyzed C–H activation reactions (which has an early history but which commanded our attention by the publication of the Murai monograph [29–30]) is also causing a revolution in how we think about the preparation of aromatic/heteroaromatic molecules. Here, conceptually similar to the complex-induced proximity effect (CIPE) [31], which is operational in DoM reactivity, chelation of heteroatom groups to transition metals may be invoked, with an important exception [32–34], to rationalize *ortho* selectivity. The aromatic ring annulative chemistry, which can be achieved by C–H-activation mediated processes, perhaps best exemplified by gold-catalyzed reactions [35–36], is astonishing and defies retrosynthetic analysis. While as yet mostly empirically derived, mechanistically inadequately defined, and practically untested, the C–H activation methodologies will compete with, supersede, and replace our existing practices in synthetic aromatic chemistry in the next decade.

Perhaps of interest to the in-depth reader of this Thematic Series are a number of other methods for the synthesis of polysubstituted aromatics which are scattered, poorly systematized, and therefore underappreciated and primed for future reviews [37–46].

What else is on the aromatic chemistry horizon? Within the area of DoM (and related metal–halogen exchange) chemistry, alternative and competitive combinational metal amide bases are rapidly appearing [47–48]. The first successful glimpses of the much sought after complementary, cleaner, regiodefined, and milder procedures to *ortho, para* versus *meta* S_E_Ar reactions are being seen in Cu-catalyzed *meta-*selective reactions [49–50]. The fleeting benzyne species, representing aromatic 1,2-dipole reactivity, is experiencing a renaissance due to milder and dependable methodologies for its generation [51–52]. Somewhat akin to S_RN_1 and, to a lesser extent VNS, the Minisci radical aromatic substitution chemistry [53] requires supplementary contemporary synthetic work in order to vigorously test its viability. The prospect of metal-catalyzed dehydrogenative conversion of cycloalkanes to aromatics as new source of aromatic feedstocks is also on the immediate horizon [54].

In this Thematic Series, you will find a fine assortment of forefront contributions in the field of manipulation and modification of aromatic compounds. Since it is a “moving” Series, my introductory comments required synchronization in order to adequately reflect the breadth of the contributions.

A cornucopia of reports, representative of the subject areas described above, demonstrates the current activity in aromatic chemistry. For example, the DoM reaction as applied to the construction of molecules of value for asymmetric synthesis is nicely presented by Rob Britton; lithiation chemistry in service of heterocyclic synthesis and modification is clearly demonstrated by the work of Jonathan Clayden, Jacques Mortier and Keith Smith; the major influence of DoM tactics are comprehensively demonstrated by the review of Marco Ciufolini; the new wave of very useful magnesiation and zincation is developed by Paul Knochel and Roberto Sanz; a hint of the potential of *meta* metalation by mixed metal/amide bases is posited by Robert Mulvey; and the application of such base combinations for the ready construction of planar chiral metacyclophanes is delightfully revealed by Donal O’Shea; striking evidence of the power of aryl metal species, derived from metal–halogen exchange, for transition metal-catalyzed cross-coupling reactions towards biaryl synthesis is furnished by Frederic Leroux; the next generation of heteroaromatic functionalization through C–H activation may be gleaned from the review of the Rouen group presented by Christophe Hoarau. From the breadth of topic coverage, you will see that the “moving” Thematic Series covers a modern “moving” field.

I hope that the taste of topics covered vis-à-vis the above generalizations will stimulate the palate for the preparation of future Thematic Series highlighting progress in synthetic aromatic chemistry. My wish is that you will find the chemistry in this Thematic Series to your interest and utility, and hence read past the graphical abstract. I thank authors and all coauthors for their meticulously prepared contributions for which it has been my privilege to serve as editor.

Kingston, August 2011

Victor Snieckus

## References

[1] Smith M B, March J (2007). Aromatic Substitution, Electrophilic. March’s Advanced Organic Chemistry.

[2] Smith M B, March J (2007). Aromatic Substitution, Nucleophilic and Organometallic. March’s Advanced Organic Chemistry.

[3] Taylor R (1990). Electrophilic Aromatic Substitution.

[4] Esteves P M, de M. Carneiro J W, Cardoso S P, Barbosa A G H, Laali K K, Rasul G, Prakash G K S, Olah G A (2003). J Am Chem Soc.

[5] Barrett A G M, Bouloc N, Braddock D C, Chadwick D, Henderson D A (2002). Synlett.

[6] Crampton M R, Knipe A C, Watts W E (2004). Nucleophilic Aromatic Substitution. Organic Reaction Mechanisms, 1999.

[7] Makosza M, Wojciechowski W (2001). Heterocycles.

[8] Clayden J, Greeves S, Warren S (2001). Organic Chemistry.

[9] Rossi R A, Pierini A B, Peñéñory A B (2003). Chem Rev.

[10] Makosza M (2010). Chem Soc Rev.

[11] Makosza M, Kwast A (2004). Eur J Org Chem.

[12] Makosza M, Lemek T, Kwast A, Terrier F (2002). J Org Chem.

[13] Joule J A, Mills K (2000). Heterocyclic Chemistry.

[14] Smith M B (2001). Organic Synthesis.

[15] Hartung C G, Snieckus V, Astruc D (2002). Modern Arene Chemistry.

[16] Macklin T, Snieckus V, Dyker G (2005). Handbook of C–H Transformations.

[17] Snieckus V (1990). Chem Rev.

[18] Dolman S J, Nyrop J L, Kuethe J T (2011). J Org Chem.

[19] Faigl F, Thurner A, Molnár B, Simig G, Volk B (2010). Org Process Res Dev.

[20] Gosselin F, Lau S, Nadeau C, Trinh T, O’Shea P D, Davies I W (2009). J Org Chem.

[21] Hickey M R, Allwein S P, Nelson T D, Kress M H, Sudah O S, Moment A J, Rodgers S D, Kaba M, Fernandez P (2005). Org Process Res Dev.

[22] Thompson A S, Corley E G, Huntington M (1996). Improved synthesis of cyclopropylacetylene. WO1996022955A1.

[23] Lo Y S, Rossano L T, Meloni D J, Moore J R, Lee Y-C, Arnett J F (1995). J Heterocycl Chem.

[24] Larsen R D, King A O, Chen C Y, Corley E G, Foster B S, Roberts F E, Yang C, Lieberman D R, Reamer R A (1994). J Org Chem.

[25] Ackermann L, Ackermann L (2009). Arylation reactions: A historical perspective. Modern Arylation Methods.

[26] Anctil E J-G, Snieckus V (2002). J Organomet Chem.

[27] Anctil E J-G, Snieckus V, Diederich F, de Meijere A (2004). Metal-Catalyzed Cross-Coupling Reactions.

[28] Torborg C, Beller M (2009). Adv Synth Catal.

[29] Murai S (1999). Activation of Unreactive Bonds and Organic Synthesis.

[30] Kakiuchi F, Murai S (2002). Acc Chem Res.

[31] Whisler M C, MacNeil S, Snieckus V, Beak P (2004). Angew Chem, Int Ed.

[32] Hartwig J F (2011). Chem Soc Rev.

[33] Maleczka R E, Shi F, Holmes D, Smith M R (2003). J Am Chem Soc.

[34] Hurst T E, Macklin T K, Becker M, Hartmann E, Kügel W, Parisienne-La Salle J-C, Batsanov A S, Marder T B, Snieckus V (2002). Chem–Eur J.

[35] Toste F D (2011). Beilstein J Org Chem.

[36] de Haro T, Nevado C (2011). Synthesis.

[37] Fringuelli F, Taticchi A (2002). The Diels–Alder Reaction.

[38] Boger D L, Weinreb S M (1987). Hetero Diels–Alder Methodology in Organic Synthesis.

[39] Bamfield P, Gordon P F (1984). Chem Soc Rev.

[40] Langer P (2009). Synlett.

[41] Castro A M M (2004). Chem Rev.

[42] Nubbemeyer U (2003). Synthesis.

[43] Clark J S, Clark J S (2002). Nitrogen, Oxygen and Sulfur Ylides: an overview. Nitrogen, Oxygen and Sulfur Ylide Chemistry.

[44] Mori K, Sato K (1982). Tetrahedron.

[45] Chandrasekaran S, Turner J V (1982). Tetrahedron Lett.

[46] Kikuchi Y, Hasegawa Y, Matsumoto M (1982). Tetrahedron Lett.

[47] Haag B A, Sämann C, Jana A, Knochel P (2011). Angew Chem.

[48] Mulvey R E (2009). Acc Chem Res.

[49] Phipps R J, Gaunt M J (2009). Science.

[50] Chen B, Hou X-L, Li Y-X, Wu Y-D (2011). J Am Chem Soc.

[51] Wenk H H, Winkler M, Sander M (2003). Angew Chem, Int Ed.

[52] Sanz R (2008). Org Prep Proced Int.

[53] Minisci F, Vismara E, Fonatana F (1989). Heterocycles.

[54] Izawa Y, Pun S, Stahl S S (2011). Science.

